# Men and Marriage

**Published:** 1887-12-31

**Authors:** 


### THE DOCTOR'S PULPIT.

### MEN AND MARRIAGE.

COMPLAINT has been made that women have often been preached at from the "Doctor's Pulpit," but that men have been passed over altogether. Some fair correspondents have done everything but ask for the use of the pulpit in order that they might have their turn and give the men a "a piece of their minds." Not one of them has, however, sent us a carefully-considered sermon on any topic specially relating to men. If they had we should have been strongly inclined to print it; because, we hold that a pulpit should be the honestest simplest and most candid public rostrum in all the world.

The natural condition of life for healthy adult men and women is marriage. The evident intention of Nature, or, as we would prefer to say, of the intelligent Creator and Ruler of mankind, is the companionship of man and woman in a life-long union. The Christian Church which, whatever may be thought of it by certain critics, is at least a venerable institution, and one which has drawn to itself some of the most noble and ingenuous natures of every age—that Church has always held marriage as one of the most sacred of human obligations, and the largest branch has exalted it to the rank of a sacrament. No doubt there are flippant persons who will brush away considerations of this kind in a moment, and will affirm that the very fact that the Church has assigned such importance to marriage is a reason why "rational people" should regard it as a mere civil contract dissoluble at the mutual convenience of the contracting parties. People with more brains and less flippancy will, however, think that an usage which is thousands of years old, and which has received such solemn sanction from many of the most venerable of all ages, deserves that deep and serious attention which was given to the great and virtuous thinkers of the past, whilst they lived, and which would be given to them again if they could all be suddenly revivified and brought before us in one grand assembly of living men. Men's thoughts lose nothing in value because those who uttered them are dead. It may be affirmed therefore that in the judgment of many of the world's ablest and most virtuous minds the natural, wholesome, healthy, and happy condition for adult men and women now, as in past ages, is marriage.

It is equally true, and, if possible, still more obvious, that marriage in this country for a very large number of men is difficult, if not practically impossible. To them the problem is of this kind: "If I marry, my wife and myself must live in a house too small for health, on an income too small for comfort, and with a prospect too dark to be contemplated with hope. Granted that I love a woman and that she loves me, the best proof I can give of my love is to avoid her, and on no account to ask her to join her lot with mine. Is it better, under such circumstances, to seek marriage or to shun it?" We answer, unhesitatingly, to shun it. But we also answer that if celibacy be decided upon, it must be a virtuous celibacy. He who decides against marriage on the score of insufficient means, and then deliberately chooses to waste a portion of his substance in vice and debauchery, is a fool of the worst kind; a fool who sins quite as much against his body as against his soul, and whom the voice of scientific medicine condemns as strongly as the voice of religion. There are no more dangerous enemies to young men than those, either in the medical profession or out of it, who encourage them to the indulgence of unlicensed passions; and no doctors are more strongly condemned by the most eminent physicians and surgeons than the dangerous few who stoop to such base methods of gaining a degrading livelihood.

The fact, however, of the practical impossibility of marriage on the part of so many men is one of serious significance, and requires the gravest consideration of all those who desire the well-being of their fellows as well as the growth of religion and virtue. It does not appear that it is a question with which legislative bodies can profitably meddle; but public teachers and those who do the thinking for their fellow-men can and ought to give serious and frequent heed to a business of such immediately practical moment. Whilst this is so, it is also certain that young men themselves, as the persons most vitally concerned, must look the situation squarely in the face. Any young man, say of four or five and twenty, who cannot now afford to marry, and sees no prospect of it in the moderately near future, should take himself seriously to task.

Is England the only country in which a man can live well and happily? Is a man's present occupation the only possible means of livelihood open to pluck and energy? We do not say he must necessarily change either his occupation or his country, and indeed so much do we think an Englishman may fairly be expected to love his fatherland that we cannot think he will contemplate leaving it without many pangs. Nevertheless, every youth should look to playing a man's part in the world; and as a rule an unmarried man does not and cannot play such a part. Moreover, the married state, in spite of many and very obvious drawbacks, is infinitely happier than the single condition; and the reward of a loving and virtuous wife is worth a large sacrifice. even one so large as voluntary and, if need be, permanent expatriation.

A good many young men, and especially those who have been born and bred in large towns, looks upon marriage as hopeless, and quietly settle themselves down to what they regard as the inevitable. That is one of the results of modern civilisation, and of the rapid growth of large towns. It is an absolutely and altogether bad result. Such young men are sorry specimens of mankind. They are without force of character, destitute of courage; and deserve nothing but contempt if they make no effort to change their condition. Do they ask what they shall do? Let them ask it by all means. But of whom should they ask it? Should they not ask themselves? Who is so much interested in a man's prosperity and happiness as himself? Why should the Prime Minister—why should the religious teacher—why should even a full-grown man's own father think more of the prosperity of his son than the son himself? Self-thought. self-purpose, self-reliance, self-help—these are the methods of vigorous, virtuous, and victorious life. If a man is to fulfil all the purposes of the life which has been bestowed upon him, he will look forward to playing his part as a husband and father just as he has played it as a son and brother, and will not be content until he has found for himself, and one gentle and loving woman, a place which may fittingly bear the sacred name of "Home." If marriage is to become the privilege of the wealthy instead of being the healthy and happy lot of all, religion and virtue will gradually become impossible, and with their decay will commence the degeneration and destruction of the State.

